# Neurological Soft Signs at Presentation in Patients With Pediatric Acute-Onset Neuropsychiatric Syndrome

**DOI:** 10.1001/jamanetworkopen.2025.0314

**Published:** 2025-03-07

**Authors:** Jane E. Zebrack, Jaynelle Gao, Britta Verhey, Lu Tian, Christopher Stave, Bahare Farhadian, Meiqian Ma, Melissa Silverman, Yuhuan Xie, Paula Tran, Margo Thienemann, Jenny L. Wilson, Jennifer Frankovich

**Affiliations:** 1 Division of Allergy, Immunology, and Rheumatology, Department of Pediatrics, Stanford University School of Medicine, Stanford, California; 2 Stanford PANS/Immune Behavioral Health Clinic and PANS Research Program at Lucile Packard Children’s Hospital, Stanford, California; 3 Department of Biomedical Data Science, Stanford University School of Medicine, Stanford, California; 4 Lane Medical Library, Stanford University School of Medicine, Stanford, California; 5 Division of Child and Adolescent Psychiatry and Child Development, Department of Psychiatry and Behavioral Sciences, Stanford University School of Medicine, Stanford, California; 6 Division of Pediatric Neurology, Department of Pediatrics, Oregon Health & Science University, Portland

## Abstract

**Importance:**

Studies of brain imaging and movements during rapid eye movement sleep indicate basal ganglia involvement in pediatric acute-onset neuropsychiatric syndrome (PANS). Characterizing neurological findings that commonly present among patients with PANS could improve diagnostic accuracy.

**Objectives:**

To evaluate the prevalence of neurological soft signs (NSSs) that may be associated with basal ganglia dysfunction among youths presenting with PANS and assess whether clinical characteristics of PANS correlate with NSSs that may be associated with basal ganglia dysfunction.

**Design, Setting, and Participants:**

This cohort study included 135 new patients who met strict PANS criteria and were evaluated at the Stanford Children’s Immune Behavioral Health Clinic between November 1, 2014, and March 1, 2020. Data on these patients were retrospectively reviewed between December 13, 2020, and September 25, 2023. Sixteen patients were excluded because they had no neurological examination within the first 3 visits and within 3 months of clinical presentation. Statistical analysis was conducted between September 26, 2023, and November 22, 2024.

**Main Outcomes and Measures:**

The following NSSs that may be associated with basal ganglia dysfunction were recorded from medical record review: (1) glabellar tap reflex, (2) tongue movements, (3) milkmaid’s grip, (4) choreiform movements, (5) spooning, and (6) overflow movements. Data from prospectively collected symptoms and impairment scales (Global Impairment Score [GIS; score range, 1-100, with higher scores indicating greater impairment] and Caregiver Burden Inventory [score range, 0-96, with higher scores indicating greater caregiver burden]) were included.

**Results:**

The study included 119 patients; the mean (SD) age at PANS onset was 8.2 (3.6) years, the mean (SD) age at initial presentation was 10.4 (3.6) years, and 66 (55.5%) were boys. At least 1 NSS that may be associated with basal ganglia dysfunction was observed in 95 patients (79.8%); the mean (SD) number of NSSs was 2.1 (1.6). Patients with 4 or more NSSs had higher GISs (mean [SD] score, 56.0 [22.6] vs 40.6 [26.7]; *P* = .05) and more symptoms (mean [SD] number, 15.1 [4.9] vs 11.5 [4.2]; *P* = .008) than patients with 0 NSSs. There was no significant difference in age at visit or in Caregiver Burden Inventory score. On Poisson and linear regression, the number of NSSs was associated with global impairment, with 1 more sign increasing the GIS by 2.86 (95% CI, 0.09-5.62; *P* = .04), and with the number of symptoms, with 1 more sign increasing the number of symptoms by 5% (1.05; 95% CI, 1.02-1.08; *P* = .002), but not with age or duration of PANS at presentation.

**Conclusions and Relevance:**

This cohort study of patients with PANS found a high prevalence of NSSs that may be associated with basal ganglia dysfunction and an association between these NSSs and disease severity that was not associated with younger age. PANS may have a unique profile, suggesting that targeted neurological examinations may support PANS diagnosis.

## Key Points

**Question:**

Do patients with pediatric acute-onset neuropsychiatric syndrome present with neurological soft signs associated with basal ganglia dysfunction?

**Findings:**

In this cohort study of 119 patients with PANS, most patients presented with at least 1 neurological soft sign associated with the basal ganglia. The number of signs was associated with global impairment and the number of PANS symptoms, consistent with basal ganglia pathologic characteristics in PANS.

**Meaning:**

This study suggests that targeted neurological examinations may help support the diagnosis of PANS.

## Teaser

This cohort study evaluates the prevalence of neurological soft signs that may be associated with basal ganglia dysfunction among youths presenting with pediatric acute-onset neuropsychiatric syndrome (PANS) and assesses whether these examination findings are associated with disease severity.

## Introduction

Pediatric acute-onset neuropsychiatric syndrome (PANS) and pediatric autoimmune neuropsychiatric disorders associated with streptococcal infections (PANDAS) are abrupt-onset neuropsychiatric disorders thought to be triggered by infection.^1,2^ Basal ganglia inflammation may be an important mechanism in these disorders, based on imaging studies demonstrating basal ganglia swelling in the acute stage,^3^ microglia activation in the caudate and putamen,^4^ and microstructural changes that are most prominent in the basal ganglia.^5,6^ Sleep studies in this patient population indicating movements during rapid eye movement sleep^7,8,9^ implicate basal ganglia pathologic characteristics. Parkinson disease is a basal ganglia disorder in which movements in rapid eye movement sleep^10,11^ and the glabellar reflex^12^ can predate clinical onset of Parkinson disease.^13,14^ These findings have also been shown in PANS, suggesting PANS is a basal ganglia disorder.

Autoantibodies targeting cholinergic interneurons in the basal ganglia have been identified^15^ that could cause imbalance between excitatory and inhibitory synaptic transmission in the basal ganglia and may contribute to the cardinal symptoms of PANS. Animal models of PANDAS demonstrate an adaptive immune response involving autoantibodies and T_H_17 cells, leading to central nervous system pathologic characteristics, including neurovascular injury, blood-brain barrier disruption, activation of microglia, and loss of excitatory synaptic proteins.^16,17,18^

PANS classification criteria require abrupt onset or abrupt recurrence of obsessive-compulsive symptoms and/or eating restrictions with 2 or more additional new and abrupt-onset neuropsychiatric symptoms, which commonly include emotional lability, irritability, aggression, severely oppositional behaviors, behavioral regression and/or behavior outbursts, deterioration in school performance, sensory amplification, motor abnormalities, sleep disturbances, and urinary issues.^19,20,21^

Although “hard” neurological examination findings of basal ganglia dysfunction, such as chorea or dystonia, suggest a condition other than PANS, neurological soft signs (NSSs), such as voluntary movement overflow, have been described in attention-deficit/hyperactivity disorder (ADHD),^22,23,24,25,26,27,28,29,30,31,32^ obsessive-compulsive disorder (OCD),^22,23,33,34,35,36,37,38^ autism,^22,23,32,39,40,41,42,43^ and Sydenham chorea^44,45,46,47^ and may also indicate basal ganglia dysfunction. The prevalence of these findings in PANS is not known.

The goal of this study was to characterize the prevalence of NSSs that may be associated with basal ganglia dysfunction and examine the association between the number of NSSs and the impairment levels at clinical presentation of PANS. We hypothesized a high prevalence of NSSs among patients with PANS and that patients with more NSSs would exhibit more severe disease states.

## Methods

### Study Setting

This was a retrospective cohort study conducted at the Stanford Children’s Immune Behavioral Health (IBH) Clinic, where children and young adults with PANS and PANDAS are treated by multiple subspecialties, including child psychiatry, rheumatology, immunology, pediatrics, and psychology. Informed written consent from parents and adult participants and written assent from competent minor participants were obtained after the nature of the research was fully described and before any data were collected. Data were stored in a secure database. Approval was given by the Stanford University institutional review board. The Strengthening the Reporting of Observational Studies in Epidemiology (STROBE) reporting guideline for cohort studies was followed.

### Study Population

The IBH Clinic saw 135 new patients between November 1, 2014, and March 1, 2020, who consented to the research and received a diagnosis of PANS (eAppendix 1 in Supplement 1) from a child psychiatrist (M.S., Y.X., P.T., or M.T.). Neurological examinations were completed as part of the physical examination by clinicians in the IBH Clinic (B.F., M.M., and J.F.). We included neurological examination findings from patients’ initial presentation to the IBH Clinic. Occasionally, at the first visit, there was no comprehensive neurological examination conducted that included NSSs that may be associated with basal ganglia dysfunction; these abridged examinations occurred for various reasons, including time restraints or patient distress. In these cases, the neurological examination from the second or third visit was used for data collection. Patients were excluded if they had no comprehensive neurological examination that included these NSSs within the first 3 visits and 3 months after the first visit (n = 16). The final study sample included 119 patients. Most examinations included (93 of 119 [78.2%]) were from the patient’s first visit to the IBH Clinic.

### Data Sources

Demographic characteristics, neurological examination findings, PANS symptoms, and impairment scale scores were abstracted from electronic medical records and prospective parent or caregiver questionnaires between December 13, 2020, and September 25, 2023. These data were maintained within our institutional review board–approved REDCap database. Race and ethnicity were self-reported. Categories included American Indian or Alaska Native, Asian, Black or African American, Jewish, Native Hawaiian or Other Pacific Islander, White, or unknown. Race and ethnicity were assessed to discern whether results are generalizable to a racial and/or ethnic population.

### Measures and Variables

Our clinical protocol was established through training with a pediatric movement disorder specialist (Terence Sanger, MD) and trainings by Susan Swedo, MD, based on her observations of the National Institutes of Health (NIH) PANDAS cohort.^48,49^ The eTable in Supplement 1 indicates the procedures and findings for each neurological test. The NSSs evaluated included (1) glabellar tap reflex, (2) abnormal tongue movements, (3) milkmaid’s grip, (4) choreiform movements, (5) spooning, and (6) overflow movements. A patient could present with a minimum of zero or a maximum of 6 NSSs that may be associated with basal ganglia dysfunction.

Prior to each clinic visit, parents and patients completed an electronic questionnaire to report interim medical or psychiatric symptoms. Impairment scales included the Global Impairment Score (GIS), a validated measure of disease severity developed for PANS,^50^ and the Caregiver Burden Inventory (CBI), a measure of caregiver burden validated for patients with PANS.^51,52,53^ The GIS ranges from 1 to 100, with higher scores indicating greater impairment, and the CBI ranges from 0 to 96, with higher scores indicating greater caregiver burden.

### Statistical Analysis

Statistical analysis was conducted between September 26, 2023, and November 22, 2024. Summary statistics were used to describe our cohort’s demographic and clinical characteristics. Continuous variables were summarized using mean (SD) values. Categorical variables were summarized using counts and proportions. Selected demographic and clinical characteristics were compared between patients with different prevalences of NSSs using 2-sample *t* tests. The following regression models were conducted, with models 1, 2, and 3 being Poisson regression and model 4 being linear regression:

- Model 1: controlling for sex and race and ethnicity, is age associated with the number of NSSs?
- Model 2: controlling for sex, race and ethnicity, and age, is PANS duration associated with the number of NSSs?
- Model 3: controlling for sex, race and ethnicity, and age, is the number of NSSs associated with the number of PANS symptoms?
- Model 4: controlling for sex, race and ethnicity, and age, is the number of NSSs associated with the GIS? (The analysis was based on 107 patients after excluding 12 patients with missing GISs.)

We conducted a sensitivity analysis by fitting the 4 models using the 71 patients who were examined for all 6 NSSs that may be associated with basal ganglia dysfunction. Model 4 sensitivity analysis was based on 67 patients after excluding 4 patients with missing GISs.

We evaluated confounders of some NSSs that could reveal an alternative explanation for the finding, such as age or comorbid conditions. We conducted 2 subset χ^2^ analyses: (1) whether or not overflow movements were associated with younger age and (2) whether or not spooning was associated with hypermobility.

All statistical tests were considered statistically significant if the 2-sided *P* < .05. We used Bonferroni correction to account for multiple testing (*P* < .025). SAS, University Edition (SAS Institute Inc), was used for analysis.

## Results

The study cohort included 119 patients with PANS (Table 1). At presentation, patients’ ages ranged from 3.9 to 22.4 years (mean [SD] age, 10.4 [3.6] years). At PANS onset, the mean (SD) age was 8.2 (3.6) years (range, 1.6-18.6 years). Patients presented to the IBH Clinic a mean (SD) of 2.2 (2.7) years (range, 0.01-11.3 years) after initial PANS onset. Most patients (105 [88.2%]) were in a disease flare at the time of evaluation. The cohort included 66 boys (55.5%) and 53 girls (44.5%), 3 Asian patients (2.5%), 14 multiracial patients (11.8%), 88 non-Hispanic White patients (73.9%), and 13 patients of other race or ethnicity (10.9%). Obsessions, compulsions, anxiety, sleep disturbance, and irritability were the most common presenting PANS symptoms. Of 29 symptoms assessed via questionnaire, patients presented with a mean (SD) of 13.2 (4.3) symptoms.

**Table 1.** Demographic and Clinical Characteristics of 119 Consecutive Patients With PANS

Characteristic	Patients with PANS (n = 119)
Age at PANS onset, mean (SD), y	8.2 (3.6)
Age at the first clinic visit, mean (SD), y	10.4 (3.6)
PANS duration, mean (SD), y	2.2 (2.7)
In PANS flare at time of evaluation, No. (%)	105 (88.2)
Sex, No. (%)	
Female	53 (44.5)
Male	66 (55.5)
Race and ethnicity, No. (%)	
Asian	3 (2.5)
Multiracial	14 (11.8)
Non-Hispanic White	88 (73.9)
Other^a^	13 (10.9)
Unknown or unreported	1 (0.8)
PANS symptoms at clinical presentation, No. (%)	
Obsessions	105 (88.2)
Compulsions	100 (84.0)
Hoarding	18 (15.1)
Food refusal or avoidance	70 (58.8)
Urge to overeat	9 (7.5)
Fluid refusal	13 (10.9)
Separation anxiety	71 (59.7)
Other anxiety, fears, phobias, or panic attacks	101 (84.9)
Emotional lability	44 (37.0)
Depression or sadness	75 (63.0)
Suicidal ideation or behavior	19 (16.0)
Irritability	84 (70.6)
Aggressive behaviors, violence, and/or rage	68 (57.1)
Oppositional behaviors	51 (42.8)
Hyperactivity or impulsivity	42 (35.3)
Trouble paying attention	67 (56.3)
Baby talk	10 (8.4)
Behavioral or developmental regression	58 (48.7)
Worsening of school performance	62 (52.1)
Worsening of handwriting, copying, or art	31 (26.0)
Cognitive impairment	70 (58.8)
Pain (headaches, abdominal pain, body pain)	78 (65.5)
Sensory dysregulation or amplification	77 (64.7)
Motor tics	43 (36.1)
Phonic tics	25 (21.0)
Daytime wetting or bedwetting (enuresis)	30 (25.2)
Urinary frequency (uses restroom frequently)	37 (31.1)
Sleep disturbance	85 (71.4)
Hallucinations	20 (16.8)
No. of above PANS symptoms, mean (SD)	13.2 (4.3)
Impairment scale scores at clinical presentation, mean (SD)	
Global Impairment Score^b^	43.2 (23.2)
Caregiver Burden Inventory^c^	32.1 (17.1)
Hypermobility at clinical presentation, No. (%)	14 (11.8)

Abbreviation: PANS, pediatric acute-onset neuropsychiatric syndrome.

^a^ Other racial and ethnic identities include American Indian or Alaska Native, Middle Eastern, and Native Hawaiian or Other Pacific Islander.

^b^ There were 12 patients (10.1%) who had missing Global Impairment Scores.

^c^ There were 44 patients (37.0%) who had missing Caregiver Burden Inventory scores.

Neurological soft signs that may be associated with basal ganglia dysfunction were common in our cohort: 71 patients (59.7%) presented with overflow movements, 62 (52.1%) with choreiform movements, 37 (31.1%) with abnormal tongue movements, 36 (30.2%) with milkmaid’s grip, 26 (21.8%) with glabellar tap reflex, and 21 (17.6%) with spooning (Table 2). These proportions were notably higher among the subset of patients who had a complete neurological examination, meaning they were examined for all 6 NSSs.

**Table 2.** Prevalence of NSSs Associated With Basal Ganglia Dysfunction for 119 Consecutive Patients With PANS at Presentation

Abnormal finding (NSSs) on examination by group	No.	Patients, No. (%)			
		Abnormal or present	Equivocal	Normal or absent	Not done or not documented
Glabellar tap reflex					
Among entire cohort	119	26 (21.8)	13 (10.9)	47 (39.5)	33 (27.7)
Among those examined	86	26 (30.2)	13 (15.1)	47 (54.6)	NA
Among those examined for all 6 NSSs	71	22 (30.9)	10 (14.1)	39 (54.9)	NA
Abnormal tongue movements					
Among entire cohort	119	37 (31.1)	11 (9.2)	50 (42.0)	21 (17.6)
Darting tongue	119	7 (5.9)	NA	NA	NA
Wormian tongue	119	29 (24.4)	NA	NA	NA
Deviation	119	10 (8.4)	NA	NA	NA
Among those examined	98	37 (37.3)	11 (11.2)	50 (51.0)	NA
Among those examined for all 6 NSSs	71	27 (38.0)	11 (15.5)	33 (46.5)	NA
Milkmaid’s grip					
Among entire cohort	119	36 (30.2)	8 (6.7)	53 (44.5)	22 (18.5)
Among those examined	97	36 (37.1)	8 (8.2)	53 (54.6)	NA
Among those examined for all 6 NSSs	71	30 (42.2)	6 (8.4)	35 (49.3)	NA
Choreiform movements					
Among entire cohort	119	62 (52.1)	7 (5.9)	47 (39.5)	3 (2.5)
Choreiform leg movements	119	6 (5.0)	NA	NA	NA
Choreiform arm movements	119	38 (31.9)	NA	NA	NA
Choreiform finger movements (“piano-playing” finger movements)	119	47 (39.5)	NA	NA	NA
Among those examined	116	62 (53.4)	7 (6.0)	47 (40.5)	NA
Among those examined for all 6 NSSs	71	45 (63.3)	6 (8.4)	20 (28.2)	NA
Spooning					
Among entire cohort	119	21 (17.6)	4 (3.4)	91 (76.5)	3 (2.5)
Among those examined	116	21 (18.1)	4 (3.4)	91 (78.4)	NA
Among those examined for all 6 NSSs	71	18 (25.3)	3 (4.2)	50 (70.4)	NA
Overflow movements					
Among entire cohort	119	71 (59.7)	3 (2.5)	37 (31.1)	8 (6.7)
In touchdown position	119	39 (32.8)	NA	NA	NA
In stressed gait	119	60 (50.4)	NA	NA	NA
Among those examined	111	71 (64.0)	3 (2.7)	37 (33.3)	NA
Among those examined for all 6 NSSs	71	51 (71.8)	1 (1.4)	19 (26.8)	NA
Among those examined at >9 y of age	69	40 (58.0)	2 (2.9)	27 (39.1)	NA

Abbreviations: NA, not applicable; NSSs, neurological soft signs; PANS, pediatric acute-onset neuropsychiatric syndrome.

All 119 patients were examined for at least 1 NSS; 95 (79.8%) presented with at least 1 NSS (Table 3), and the mean (SD) number of NSSs was 2.1 (1.6). Among those examined for all 6 NSSs (71 [59.7%]), 91.5% (65 of 71) presented with at least 1 NSS, and the mean (SD) number of NSSs was 2.6 (1.4) (Figure).

**Table 3.** Comparison of Age, PANS Duration, and Disease Severity at Presentation Between Patients With and Patients Without NSSs Associated With Basal Ganglia Dysfunction

Characteristic	No. of NSSs (No./total No. of patient [%])		
	0 (24/119 [20.2%])	≥1 (95/119 [79.8%])	≥4 (26/119 [21.8%])
Age at the first clinic visit, mean (SD), y	10.4 (4.8)	9.7 (3.2)	9.8 (2.6)
PANS duration, mean (SD), y	3.2 (3.3)	1.9 (2.5)^a^	1.5 (2.5)^a^
No. of PANS symptoms, mean (SD)	11.5 (4.2)	13.6 (4.3)^a^	15.1 (4.9)^b^
Global Impairment Score, mean (SD) [No.]	40.6 (26.7) [n = 19]	43.8 (22.4) [n = 88]	56.0 (22.6) [n = 22]^c^
Caregiver Burden Inventory score, mean (SD) [No.]	32.2 (18.4) [n = 13]	32.1 (17.0) [n = 62]	32.0 (17.2) [n = 17]

Abbreviations: NSSs, neurological soft signs; PANS, pediatric acute-onset neuropsychiatric syndrome.

^a^ *P* < .05 (compared with patients with no NSSs).

^b^ *P* < .01 (compared with patients with no NSSs).

^c^ *P* = .05 (compared with patients with no NSSs).

**Figure.**
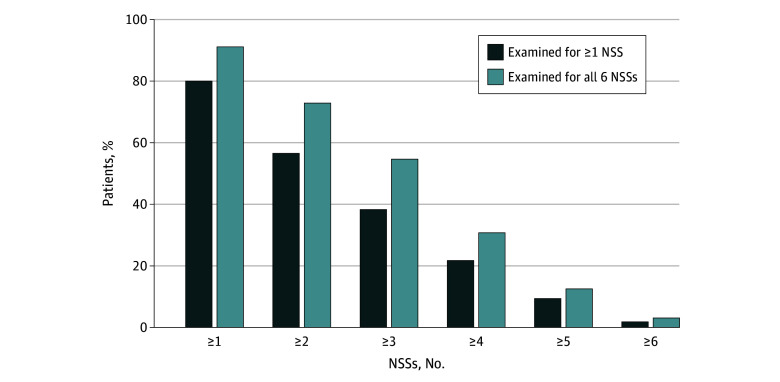
Number of Neurological Soft Signs (NSSs) Associated With Basal Ganglia Dysfunction in 119 Consecutive Patients With Pediatric Acute-Onset Neuropsychiatric Syndrome at Presentation All 119 patients were examined for at least 1 NSS, and 71 patients were examined for all 6 NSSs. Neurological soft signs associated with basal ganglia dysfunction include glabellar tap reflex, abnormal tongue movements, milkmaid’s grip, choreiform movements, spooning, and overflow movements.

Compared with the 24 patients with no NSSs, the 95 patients with 1 or more NSSs and the 26 patients with 4 or more NSSs had more PANS symptoms (0 vs ≥1 NSS: mean [SD], 11.5 [4.2] vs 13.6 [4.3] symptoms [*P* = .04]; 0 vs ≥4 NSSs: mean [SD], 11.5 [4.2] vs 15.1 [4.9] symptoms [*P* = .008]) and shorter PANS duration (0 vs ≥1 NSS: mean [SD], 3.2 [3.3] vs 1.9 [2.5] years [*P* = .03]; 0 vs ≥4 NSSs: mean [SD], 3.2 [3.3] vs 1.5 [2.5] years [*P* = .04]) (Table 3). Compared with patients with no NSSs who provided GISs (13 of 24), patients with 4 or more NSSs who provided GISs (22 of 26) reported worse global functioning of borderline significance (mean [SD] GIS, 56.0 [22.6] vs 40.6 [26.7]; *P* = .05). These results did not survive the Bonferroni correction (*P* = .003). There was no significant difference in age or CBI score.

On Poisson regression, neither age at first visit nor PANS duration was associated with the number of NSSs (models 1 and 2). Model 3 showed that the number of NSSs was significantly associated with the number of PANS symptoms, with 1 more sign increasing the number of symptoms by 5% on average (1.05; 95% CI, 1.02-1.08; *P* = .002; Bonferroni-adjusted *P* = .04). On linear regression, model 4 showed that the number of NSSs was significantly associated with GIS, with 1 more sign increasing the GIS by 2.86 points on average (2.86; 95% CI, 0.09-5.62; *P* = .04). This finding was not significant after adjusting for multiple testing (Bonferroni-adjusted *P* = .08).

In a sensitivity analysis using the 71 patients examined for all 6 signs, neither age at first visit nor PANS duration was associated with the number of NSSs that may be associated with basal ganglia dysfunction. The number of NSSs was significantly associated with the number of PANS symptoms, with 1 more sign increasing the number of symptoms by 6% on average (1.06; 95% CI, 1.02-1.10; *P* = .001; Bonferroni-adjusted *P* = .02). The number of NSSs was significantly associated with the GIS, with 1 more sign increasing the GIS by 5.19 points on average (5.19; 95% CI, 1.96-8.43; *P* = .002; Bonferroni-adjusted *P* = .04). Because patients can have up to 6 NSSs, the estimated association with symptoms and GIS per NSS is significant.

There was no significant association found between overflow movements and being 9 years of age or younger (patients aged ≤9 years, 31 of 42 [73.8%]; patients aged >9 years, 40 of 69 [58.0%]) (Table 2). There was also no significant association found between spooning and joint hypermobility.

## Discussion

To our knowledge, this is the first study to describe the prevalence of NSSs which may be associated with basal ganglia dysfunction among patients with PANS at clinical presentation. We observed a high frequency of NSSs among patients with PANS who presented to the Stanford Children’s IBH Clinic, with more NSSs associated with more symptoms and greater impairment.

Previous studies have reported neurological findings among patients with PANS at the time of magnetic resonance imaging^6^ as well as during evaluation at the NIH.^49^ These studies are fairly consistent with our findings (Table 4).^6,27,28,29,30,31,32,36,37,38,40,41,42,43,44,45,46,47,49,54,55,56,57,58,59,60,61^ The slightly higher rates of NSSs in previous studies could be explained by selection bias; those cohorts are representative of more severe cases (only a small fraction of patients with PANS receive imaging or specialized care from the NIH program).

**Table 4.** Rates of Neurological Soft Signs Associated With Basal Ganglia Dysfunction in Populations With Developmental and Psychiatric Conditions

Abnormal findings	Typically developing youths	Youths with ADHD	Adults with OCD (no data available for youths)	Youths with ASD	Youths with SC	Youths with PANS	
						From this study	Severe enough to warrant brain MRI or NIH evaluation
Glabellar tap reflex	Not expected past full-term age^54^ On average, normal^a^ (mean score, 0.4) at age 7-12 y (mean age, 10 y)^27^	On average, mild^a^ (mean score, 1.1) at age 7-12 y (mean age, 10 y)^27^^,^^b^	On average, normal^a^ (tic-free OCD and tic-related OCD mean score, 0.3) (mean age, 28 y)^37^	19% Abnormal^c^ at age 2-25 y (mean age, 8 y)^41^	NA	21.8% Mild	26.0% Mild^6^
Motor impersistence	Not expected and rarely observed past age 7 y^55^ 3% Abnormal^d^ (score 2) and 0% maximally abnormal (score 3) at age 11-14 y (mean age, 13 y)^56^ 16% Abnormal^e^ at age 6-17 y (mean age, 11 y)^40^	67% Abnormal^f^ at age 6-15 y (mean age, 10 y)^28^	2.4% Abnormal^c^ at age 18-53 y (mean age, 34 y)^36^	27% Abnormal^e^ at age 6-17 y (mean age, 11 y)^40^ On average, mildly abnormal^a^ on posture maintenance (HiAD mean score, 1.5; LoAD mean score, 1.4) at age 7-9 y (mean age, 9 y)^42^	42.1% Milkmaid’s grip at age 3-13 y (mean age, 10 y)^44^ 18.5% Darting tongue and 26.2% milkmaid’s grip at age 6-17 y (mean age, 12 y)^46^	30.2% Mild milkmaid’s grip 24.4% Mild tongue impersistence (wormian tongue)	35.0% Mild tongue impersistence (wormian tongue)^6^
Choreiform movements	Not expected and rarely observed past age 7 y^55,57^ On average, absent^a^ (mean score, 0.4) at age 6-16 y (mean age, 11 y)^29^	On average, mild^a^ (mean score, 1.2) at age 6-16 y (mean age, 11 y)^29^^,^^b^	31.7% Abnormal^c^ at age 18-53 y (mean age, 34 y)^36^^,^^b^ 33.3% With “choreiform syndrome” in pediatric dataset^38^	On average, absent^a^ (HiAD mean score, 0.2; LoAD mean score, 0.3) at age 7-9 y (mean age, 9 y)^42^	100% Abnormal (26.3% mild chorea, 52.6% moderate chorea, 21.0% severe chorea) at age 8-16 y (mean age, 12 y)^45^	52.1% Mild	53.0% Mild,^6^ 96.2% abnormal^g^ (26.9% minimal, 19.2% moderate, 50.0% marked)^49^
Spooning	Unknown^58^	On average, not present^a^ (mean score, 0.27) at age 6-11 y (mean age, 9 y)^30^	NA	NA	22.2% Present^a^ age 6-13 y (mean age, 10 y)^47^	17.6% Mild	NA
Overflow movements	Normal in children under age 6 y^59^ and gradually disappears at ages 7-14 y,^55^ with a significant decrease at age 9 y^60,61^ 4% On stressed gait at age 6-18 y (mean age, 9 y)^31^ On average, 8 abnormal movements^h^ at age 8-12 y (mean age, 10 y)^43^ On average, 2 abnormal movements^h^ at age 8-15 y (mean age, 12 y)^32^	75% On stressed gait at age 6-19 y (mean age, 8 y)^31^^,^^b^ 93% On stressed gait at age 6-15 y (mean age, 10 y)^28^ On average, 8 abnormal movements^h^ at age 8-15 y (mean age, 10 y)^32^^,^^b^	On average, absent^a^ (tic-free OCD and tic-related OCD mean score, 0.3) (mean age, 28 y)^37^	AS: On average, 10 abnormal movements^h^ at age 8-12 y (mean age, 10 y)^43^ HFA: On average, 4 abnormal movements^h^ at age 8-15 y (mean age, 11 y)^32^	NA	59.7% Mild	12.0% Mild^6^

Abbreviations: ADHD, attention-deficit/hyperactivity disorder; AS, Asperger syndrome; ASD, autism spectrum disorder; HFA, high-functioning autism; HiAD, high-IQ autistic disorder; LoAD, low-IQ autistic disorder; MRI, magnetic resonance imaging; NA, no available data; NIH, National Institutes of Health; OCD, obsessive-compulsive disorder; PANS, pediatric acute-onset neuropsychiatric syndrome; SC, Sydenham chorea.

^a^ 0 = No abnormality, 1 = mild but definite impairment, 2 = marked impairment.

^b^ Statistically significant difference from healthy controls in referenced study.

^c^ Rated as normal or abnormal.

^d^ 0 = Absent, 3 = maximum deviation.

^e^ Abnormal = maintain arm extension and tongue protrusion for less than 40 seconds.

^f^ Abnormal = maintain eyes closed for less than 20 seconds.

^g^ 0 = No movements observed; minimal = occasional small-amplitude movements of fingers; moderate = continuous, small-amplitude movements of fingers, wrists, and proximal areas (arms or shoulders); marked = continuous, moderate-amplitude movements of fingers, wrists, and proximal areas.

^h^ Abnormal movements = total unintended movements on stressed gaits, tandem gaits, and timed movements.

Neurological soft signs are subtle examination findings thought to indicate abnormalities in neurological circuit development.^62^ These are contrasted with “hard neurological signs,” such as chorea and dystonia, which are often associated with structural and/or genetic basal ganglia abnormalities.^63^ However, distinction on examination is not always clear; choreiform movements may be a mild form of chorea, and motor overflow may be a mild form of dystonia. This has clinical implications. For example, in a child with acquired chorea without genetic findings, Sydenham chorea is the probable diagnosis. However, if the chorea is more subtle (“choreiform”), the child is more likely to have PANS, assuming PANS criteria are met. Moreover, research classification and enrollment in trials are challenging in borderline cases.

Based on a PubMed search (eAppendix 2 and eReference in Supplement 1), NSSs that may be associated with basal ganglia dysfunction are variably seen in developmental and psychiatric conditions, including ADHD, OCD, autism, schizophrenia, and Sydenham chorea (Table 4).^6,27,28,29,30,31,32,36,37,38,40,41,42,43,44,45,46,47,49,54,55,56,57,58,59,60,61^ Neurological soft signs that may be associated with basal ganglia dysfunction are typically not observed among school-aged children, with the exception of overflow movements, which are normal among children younger than 6 years^64^ and gradually disappear at 7 to 14 years of age,^65^ with a significant decrease at 9 years of age.^55,59^ Younger age was not associated with more NSSs in our study, confirming that the high prevalence of NSSs is not associated with early developmental stage.

Similar to what is reported in the literature about typically developing youths,^55,59^ in our cohort, patients 9 years of age or younger had a higher frequency of overflow movements compared with patients older than 9 years (74% vs 58%). However, this difference was not statistically significant. Moreover, the rates of overflow movements in both the younger and older subset of patients are notably high compared with the expected rates among typically developing youths (Table 4), confirming the validity of evaluating this finding among patients with PANS, especially those older than 9 years.

Although there are more than 70 NSSs and more than 160 examination maneuvers,^60^ the maneuvers in this study were chosen because PANS is a suspected basal ganglia inflammatory disorder,^3,4,5,6,7,8,9^ and these maneuvers are more likely to be associated with basal ganglia dysfunction. However, the pathophysiology is likely more complex, and other circuits may be involved. For example, in the glabellar tap sign, the basal ganglia and interconnected cortical structures have a defined role in the timing and reciprocity of blinking. Thus, persistent blinking may reflect basal ganglia dysfunction and/or frontal lobe dysfunction.^54,61^ Inability to maintain tongue protrusion and milkmaid’s grip are both manifestations of the motor impersistence observed in chorea. Tongue deviation could be due to dystonia, but it can also be observed in tongue weakness or structural abnormalities.^56,57^ However, in our patients with tongue deviation (n = 10), there were no structural changes or tongue atrophy, and tongue deviation improved as the disease flare resolved. Overflow movements are involuntary posturing in body regions outside of the area of intentional movement and may be secondary to pathologic findings in the basal ganglia, cerebellum, and sensorimotor cortex.^58^ Given the preponderance of data implicating basal ganglia involvement in PANS,^3,4,5,6,7,8,9^ our proposed set of NSSs likely reflect abnormalities in circuits involving the basal ganglia. It is possible that preexisting basal ganglia dysfunction predated and predisposed to PANS. Thus, the association between NSSs and the clinical state should be explored. Neurological soft signs that may be associated with basal ganglia dysfunction resolving with patient recovery would contradict basal ganglia involvement being a static predisposing factor.

Some clinicians have theorized that spooning is a manifestation of joint hypermobility rather than dystonic posturing, given the high prevalence of hypermobility among patients with PANS.^66,67^ In our cohort, there was no significant association between spooning and joint hypermobility, suggesting it may be a relevant dystonic feature in PANS. Nonetheless, future studies should define whether spooning is associated with other NSSs associated with basal ganglia dysfunction. More broadly, the ability to distinguish joint or muscle symptoms from subtle neurological findings should be further explored in this patient population.

In the imaging study,^6^ patients with choreiform movements at the time of magnetic resonance imaging had more diffuse brain abnormalities. In synthesis with our study, patients with PANS who present with NSSs may have not only greater inflammation but also greater disease severity. Thus, NSSs may aid diagnosis and clinical monitoring of PANS, which currently has no reliable biomarker. Because 84% of youths with PANS undergo relapses and remissions,^68^ determining the clinical state is crucial to managing symptoms and treatment. Most patients in our study (105 [88.2%]) were in a disease flare during examination at presentation, so the study may not have been powered for assessing the association between NSSs and disease state. Further study is needed to understand the evolution of NSSs associated with basal ganglia dysfunction during the course of disease.

Some NSSs may help differentiate between developmental and psychiatric conditions. For example, choreiform movements are typically absent in youths with autism^42^ but are commonly present in youths with PANS,^6,49^ ADHD,^29^ OCD,^38^ and Sydenham chorea.^45^ Thus, if a patient with a diagnosis of autism exhibits choreiform movements, further diagnostic workup for other developmental and psychiatric conditions may be warranted. Because many NSSs observed in patients with PANS are also observed in patients with ADHD, OCD, autism, and Sydenham chorea (Table 4), the presence of a specific NSS may not distinguish PANS from these other conditions. However, these conditions could have unique NSS profiles that could aid in diagnosis. This possibility underscores the significance of completing and analyzing neurological examinations in these pediatric populations. Moreover, because NSSs are often general markers of psychopathologic findings, they may help determine patients’ neuropsychiatric status, regardless of diagnosis.

### Limitations

There are some limitations to this study. Although the neurological examination was standardized, variability in methods and findings may have occurred between clinicians, and some data were missing. We were unable to conduct a comprehensive neurological examination including NSSs at every patient’s first visit, and examinations were sometimes performed during remission or resolution of a disease flare. Fourteen patients (11.8%) had their NSSs evaluated for the first time when they were not in a flare state. A challenge with this patient population is that patients who are doing poorly from a behavioral or mental health standpoint may not be able to cooperate with neurological examinations but may exhibit high levels of basal ganglia dysfunction. Thus, we may have underestimated the true prevalence of NSSs associated with basal ganglia dysfunction among patients with PANS. Last, the prevalence of NSSs among healthy youths and youths with OCD, ADHD, autism, and Sydenham chorea is not well established, so it is unclear if NSSs can distinguish PANS from other pediatric conditions. Future studies should use blinded reviews of examination videos to define the rates of NSSs associated with basal ganglia dysfunction among patients with PANS and other developmental and psychiatric conditions, as well as healthy youths.

## Conclusions

In this cohort study, children and young adults presenting with PANS commonly had multiple NSSs associated with basal ganglia dysfunction on physical examination, with more NSSs assocated with greater impairment. Although diagnosis of PANS is based primarily on history, these findings suggest that targeted neurological examinations may support diagnosis. Furthermore, the high frequency of NSSs in PANS provides further evidence that pathogenic mechanisms of PANS may involve the basal ganglia, which could be the target of psychotherapeutic medications and rehabilitation. As these examinations are seldom routinely performed by neurologists or pediatricians, we advocate for their use in evaluating youths for immune-mediated neuropsychiatric deterioration.

## Notes

Supplement 1. **eAppendix 1.** Criteria for Diagnosis of PANS **eTable.** Maneuvers Used to Evaluate Neurological Soft Signs Reflective of Basal Ganglia Dysfunction in PANS **eAppendix 2.** PubMed Search Strategy
eReference.

Supplement 2.
Data Sharing Statement
