# Supplementary material for: Neurological Soft Signs at Presentation in Patients With Pediatric Acute-Onset Neuropsychiatric Syndrome
Source: JAMA Netw Open. 2025 Mar 7;8(3):e250314. doi: 10.1001/jamanetworkopen.2025.0314 (PMC11889471; doi:10.1001/jamanetworkopen.2025.0314)
Supplement: Supplement 1. — eAppendix 1. Criteria for Diagnosis of PANS eTable. Maneuvers Used to Evaluate Neurological Soft Signs Reflective of Basal Ganglia Dysfunction in PANS eAppendix 2. PubMed Search Strategy eReference. [file jamanetwopen-e250314-s001.pdf]

Supplementary Online Content

Zebrack JE, Gao J, Verhey B, et al. Neurological soft signs at presentation in patients with pediatric acute-onset neuropsychiatric syndrome. *JAMA Netw Open.* 2025;8(3):e250314. doi:10.1001/jamanetworkopen.2025.0314

eAppendix 1. Criteria for Diagnosis of PANS
eTable. Maneuvers Used to Evaluate Neurological Soft Signs Reflective of Basal Ganglia Dysfunction in PANS
eAppendix 2. PubMed Search Strategy
eReference.

This supplementary material has been provided by the authors to give readers additional information about their work.

eAppendix 1. Criteria for Diagnosis of PANS[1]

1. Abrupt, dramatic onset of obsessive-compulsive disorder or severely restricted food intake
2. Concurrent presence of additional neuropsychiatric symptoms, with similarly severe and acute onset, from at least two of the following seven categories:
    a. Anxiety
    b. Emotional lability and/or depression
    c. Irritability, aggression and/or severely oppositional behaviors
    d. Behavioral (developmental) regression
    e. Deterioration in school performance
    f. Sensory or motor abnormalities
    g. Somatic signs and symptoms, including sleep disturbances, enuresis or urinary frequency
3. Symptoms are not better explained by a known neurologic or medical disorder, such as Sydenham chorea, systemic lupus erythematosus, Tourette disorder or others.

© 2025 Zebrack JE et al. *JAMA Network Open.*

eTable. Maneuvers Used to Evaluate Neurological Soft Signs Reflective of Basal Ganglia Dysfunction in PANS

Test	Description	Normal finding	Abnormal findings
Glabellar tap	Repetitively tap the patient's glabella (between the eyebrows)	Habituation (cessation of blinking within 5 taps)	Glabellar tap reflex (blinking persists with prolonged tapping ($\geq$5 taps))
Tongue protrusion	Ask patient to maintain their tongue in protrusion	No tongue movement, tongue remains protruded	Wormian movements of the tongue
			Darting tongue (motor impersistence)[a]
			Tongue deviation (right or left)
Grip maintenance	Ask patient to continuously squeeze the examiner's finger	Sustained muscle contraction	Milkmaid's grip (motor impersistence)[a]
Arm extension position	Ask patient to sit/stand with eyes closed and arms extended forward from shoulders	No involuntary movements	Choreiform leg movements ("chorea minima")[b]
			Choreiform arm movements ("chorea minima")[b]
			Choreiform finger movements ("piano-playing" finger movements)[b]
			Finger spooning[c]
Touchdown position	Ask patient to extend arms above head with elbows in extension and palms facing each other	No involuntary movements	Involuntary arm posturing (overflow movements)
Stressed gait	Ask patient to walk on tiptoes, on heels, and on lateral edge of feet	No involuntary movements	Involuntary arm posturing (overflow movements)

[a]motor impersistence refers to an inability to maintain consistent muscle contraction
[b]choreiform movements refer to random, irregular, involuntary subtle twitches in legs, arms, or fingers that are not as pronounced as chorea
[c]finger spooning refers to dystonic hyperextension of the fingers

© 2025 Zebrack JE et al. *JAMA Network Open.*

eAppendix 2. PubMed Search Strategy

1. ("Autism Spectrum Disorder"[Mesh] OR "Obsessive-Compulsive Disorder"[Mesh] OR "obsessive compulsive" [tw] OR austis* [tw])
2. ("dyskinesias" [mesh] OR "chorea" [mesh] OR "neurologic examination" [mesh] OR choreiform [tw] OR "soft sign*" [tw] OR glabellar [tw] OR spooning [tw] OR overflow [tw] OR milkmaid [tw] OR tongue [tw] OR (movement [tw] AND impersist* [tw]))
3. (infan* [tw] OR newborn* [tw] OR "new-born" [tw] OR "new borns" [tw] OR perinat* [tw] OR neonat* [tw] OR baby [tw] OR baby* [tw] OR babies [tw] OR toddler* [tw] OR minors [tw] OR child [tw] OR child* [tw] OR children* [tw] OR schoolchild* [tw] OR "school child" [tw] OR "school children" [tw] OR adolescen* [tw] OR juvenil* [tw] OR youth* [tw] OR teen* [tw] OR "under age" [tw] OR pubescen* [tw] OR prepubesc* [tw] OR pediatric* [tw] OR paediatric* [tw] OR "Nursery school" [tw] OR kindergar* [tw] OR "primary school" [tw] OR "secondary school" [tw] OR "elementary school" [tw] OR "high school" [tw] OR "primary schools" [tw] OR "secondary schools" [tw] OR "elementary school" [tw] OR "high schools" [tw] OR highschool* [tw] OR "infant" [mesh] OR "Child"[mesh] OR "Adolescent"[mesh] OR "Minors" [mesh] OR "Puberty" [mesh] OR "Pediatrics" [mesh] OR pediatrics) AND english [lang]
4. 1 AND 2 AND 3

© 2025 Zebrack JE et al. *JAMA Network Open.*

eReference.

1. Swedo SE, Leckman JF, Rose NR. From Research Subgroup to Clinical Syndrome: Modifying the PANDAS Criteria to Describe PANS (Pediatric Acute-onset Neuropsychiatric Syndrome). *Pediatrics & Therapeutics.* 2012;2(2):1-8. doi:10.4172/2161-0665.1000113

© 2025 Zebrack JE et al. *JAMA Network Open.*
