# Supplementary material for: Neurological Soft Signs at Presentation in Patients With Pediatric Acute-Onset Neuropsychiatric Syndrome
Source: JAMA Netw Open. 2025 Mar 7;8(3):e250314. doi: 10.1001/jamanetworkopen.2025.0314 (PMC11889471; doi:10.1001/jamanetworkopen.2025.0314)
Supplement: Supplement 2. — Data Sharing Statement [file jamanetwopen-e250314-s002.pdf]

# Data Sharing Statement

Zebrack. Neurological Soft Signs at Presentation in Patients With Pediatric Acute-Onset Neuropsychiatric Syndrome. *JAMA Netw Open*. Published March 07, 2025. doi:10.1001/jamanetworkopen.2025.0314

**Data**
**Data available:** Yes
**Data types:** Deidentified participant data
**How to access data:** Contact jfranko@stanford.edu for deidentified participant data.
**When available:** With publication

**Supporting Documents**
**Document types:** None

**Additional Information**
**Who can access the data:** Researchers whose proposed use of the data has been approved by their institutional IRB and our Stanford IRB
**Types of analyses:** For purposes approved by their institutional IRB and our Stanford IRB
**Mechanisms of data availability:** With a signed data access agreement
